# Mass media campaigns to reduce unnecessary caesarean sections: a systematic review

**DOI:** 10.1136/bmjgh-2019-001935

**Published:** 2020-02-26

**Authors:** Maria Regina Torloni, Vanessa Brizuela, Ana Pilar Betran

**Affiliations:** 1 Evidence Based Healthcare Postgraduation Program, Department of Medicine, Universidade Federal de São Paulo, Sao Paulo, Brazil; 2 UNDP/UNFPA/UNICEF/WHO/World Bank Special Programme of Research, Development and Research Training in Human Reproduction (HRP), Department of Sexual and Reproductive Health and Research, World Health Organization, Geneva, Switzerland

**Keywords:** health education and promotion, maternal health, obstetrics, systematic review

## Abstract

**Introduction:**

The worldwide increase in unnecessary caesarean sections (CSs) is a major global health issue. Mass media campaigns have been used in several countries to reduce this trend. The objectives of this systematic review were to identify, critically appraise and synthesise the findings, including the barriers and enablers, of mass media campaigns directed at lay people to reduce unnecessary CS.

**Methods:**

We included any study design that reported health communication mass media campaigns directed at lay people with the specific objective of reducing unnecessary CS, created by any agent, in any format. We searched seven electronic databases without language restrictions, from inception to February 2019. Experts in the field were contacted.

**Results:**

The search yielded 14 320 citations; 50 were selected for full-text reading; and one was included. Six other reports were included. The seven campaigns were conducted in 2009–2017, mostly in Latin America. Most campaigns were independent efforts by non-governmental or activist organisations. Only one campaign conducted formative research and pretested the intervention. All campaigns used indirect communication, mostly through internet channels; two campaigns also used direct communication with the public. None assessed their effects on CS rates. Only two campaigns measured their impacts on participants’ knowledge, attitudes and birth preferences but only in the short term. The main barriers were lack of financial and human resources. The main enablers were the enthusiasm of volunteers, the participation of famous persons/celebrities and the involvement of communication professionals.

**Conclusions:**

There are few mass media campaigns directed at lay people to reduce CS. Most campaigns did not use key principles recommended for the creation and implementation of health communication interventions, and none assessed their effects in reducing CS rates. If media campaigns can play a role in modifying population views towards CS, there is a need for more rigorous studies including impact assessment.

**PROSPERO registration number:**

CRD42019120314.

**Key messages**

**What is already known?**

- Scientific evidence does not support caesarean sections (CSs) for non-medical reasons, and numerous studies report increased maternal and perinatal risks associated with this type of delivery.
- The worldwide overuse of CS is a major global health issue that has led to considerable international debate.
- Mass media campaigns are recommended to change population health behaviours, but there are no systematic reviews on campaigns to reduce CS rates.

**What are the new findings?**

- We found four campaigns in Latin America, two in Europe and one in Asia directed at lay people with the specific objective of reducing unnecessary CS.
- Most campaigns were independent efforts by non-governmental or activist organisations, and did not use key principles recommended for the creation and implementation of health communication interventions.
- None assessed their effects on CS rates.

**What do the new findings imply?**

- There is a paucity of publications and data about mass media campaigns to reduce unnecessary CS.
- If mass media campaigns can play a role in modifying population views towards CS, there is a need for more rigorous studies including impact assessment.

## Introduction

During the last decade, there has been a steady worldwide increase in the rate of caesarean sections (CSs). In 2014, the global CS rate reached 19.1%, a threefold increase from 1990, with large variations between and within countries. Currently, Latin America and the Caribbean have the highest CS rates (40.5%), followed by North America (32.3%), Oceania (31.1%), Europe (25%), Asia (19.2%) and Africa (7.3%).^1 2^ When performed for medical indications, a CS is a valuable intervention that can save the lives of mothers and babies, or avoid serious complications that can have long lasting consequences. However, scientific evidence does not support CS for non-medical reasons, and there are numerous reports of increased maternal and perinatal risks associated with this type of delivery.^3–8^

Maternal request, motivated by convenience, fear, misconceptions or cultural trends, is often mentioned as one of the key contributors to the increase in CS rates.^9–18^ The attitudes, beliefs and behaviours of adults are determined by numerous factors and can be influenced by various communication channels and the media.^19 20^ Reviews have shown the effectiveness of mass media campaigns targeted at reducing unhealthy habits or promoting healthy behaviours such as breast feeding or family planning, among others.^21–27^ However, significant changes in behaviour can be achieved only when campaigns are well designed and carried out at sufficient scale and intensity.^21 28–36^

Over the last decade, there have been some campaigns targeted at the general population to reduce unnecessary CS. In view of the increasing concern about the worldwide rise in caesarean deliveries, potential negative consequences of CS for the health of mothers and infants, and the limited effectiveness of strategies tested to date to revert this trend,^37 38^ it is important to investigate the main characteristics, barriers and enablers, and the impact of these campaigns. However, to the best of our knowledge, up to the present, there has been no systematic review of these campaigns. This information gap motivated us to perform this review.

The objectives of this review were to identify, critically appraise and synthesise the information available on mass media campaigns directed at lay people to reduce unnecessary CSs. We assessed the main characteristics, the effectiveness, and the barriers and enablers to the creation and implementation of these campaigns.

## Methods

This review followed Cochrane methods and the MOOSE reporting recommendations.^39 40^ The protocol of the review was registered in the International Prospective Register of Systematic Reviews database.

### Selection criteria

#### Types of studies

We included any type of report on mass media campaigns directed at lay people to reduce unnecessary CS or to increase vaginal delivery (VD). This included articles published in scientific journals or presented in events, as well as written, audio or video material available on the internet (including YouTube) or on social media channels (Facebook, Instagram and others). We included randomised, quasi-randomised, cluster randomised or non-randomised trials, as well as controlled before–after studies or interrupted time series studies or case reports. The reports had to describe the main components of the campaign in sufficient detail so that they could be understood and eventually adapted/replicated in other settings. We included only reports that stated that the main objective (or primary outcomes) of the campaign was to reduce unnecessary CS or to increase VD.

#### Types of campaigns (exposure)

We defined a mass media campaign as an intervention that uses a set of organised communication activities to produce specific results or effects in a relatively large number of individuals, usually within a specific period of time.^41 42^ We included campaigns created or promoted by any agent, such as local or federal government authorities, national or international governmental or non-governmental organisations (NGOs), or specific associations/groups of individuals. We excluded messages created and posted/disseminated by single individuals or small groups (e.g., an activist promoting messages against CS in his/her blog or Facebook page), as these do not fulfil the classic definition of a campaign. We included campaigns whose main messages were the reduction of primary or repeat CS or the promotion of VD in otherwise healthy nulliparous or multiparous women. Campaigns using any type of communication channel were eligible. These included (1) written material disseminated through pamphlets, newspapers, magazines, mobile phones, billboards or posters; (2) audio messages disseminated on radio, television (TV) or phone (recorded messages); and (3) any form of material disseminated over the internet and social media (YouTube, Twitter, Facebook and Instagram).

We included campaigns that used either direct or indirect communication strategies. Direct communication strategies involve the transmission of messages through face-to-face interventions such as presentations, performances and group discussion sessions in clinics, schools or other public areas. Indirect communication campaigns use mass media instruments such as radio, TV, movies, newspapers or magazines to transmit their message to the general public during a specific period of time. This mode of communication is indirect because the consumer was not expecting this information, and it is presented to him/her in the context of another experience of his/her interest.^42–44^

We included campaigns of any cost, size, duration or catchment area conducted as a stand-alone intervention or as part of a broader, multicomponent, intervention. We included campaigns with or without formative research to inform the creation and development of the campaign, as well as campaigns with or without previous research to assess the baseline and/or postintervention (before and after the campaign) outcomes of interest.

#### Types of participants

We included studies that described campaigns targeted at healthy pregnant women (of any parity, with or without previous CS), non-pregnant women of reproductive age or the general lay public (men and women of any age). We included campaigns targeted at laypersons of any nationality, gender, social class or educational level. We excluded campaigns aimed at specific high-risk groups, such as people with HIV, as well as campaigns targeted exclusively at health professionals or institutions.

#### Types of outcomes

We included reports with or without quantitative assessments of the impact of the campaign on hard or soft outcomes. Hard outcomes were changes in the rates of CS or VD after the campaign, or in the exposed versus non-exposed groups. Soft outcomes were changes in knowledge, attitudes, beliefs or preferences for route of delivery on a sample or all participants after the campaign or in exposed versus non-exposed groups.

We collected and present the main barriers and enablers described by authors for the creation and implementation of their campaigns as part of the outcomes of this review.

### Search strategy

The search strategy used the following general search words, adapted to each specific database: ‘campaigns’, ‘birth’ and ‘preferences’ (see online supplementary file 1 for the full search strategy). We ran the search in the following electronic databases, from inception until 1 February 2019, without language restrictions: MEDLINE (via PubMed), Embase (via Ovid), CINHAL, PsycINFO, Soclit, Web of Science, Popline, Global Index Medicus (which includes LILACS, AIM, IMSEAR, WPRIM and IMEMR) and EBSCO Multidatabase (which includes 37 databases). We also searched the website of the Center for Communication Programs (https://ccp.jhu.edu/). We screened the reference lists of all studies selected for full-text reading and contacted experts in the field to identify additional potentially relevant campaigns.

10.1136/bmjgh-2019-001935.supp1 Supplementary data

### Process of study selection and data extraction

All citations identified from electronic databases were uploaded into Covidence (Veritas Health Innovation, Melbourne, Australia). After the exclusion of duplicates, two investigators independently screened the titles and abstracts of all citations to select potentially relevant studies. The full texts of these studies were retrieved and read; those that fulfilled the aforementioned selection criteria were included in the review. Discrepancies between reviewers were discussed until consensus was reached; if needed, a third reviewer was invited to arbitrate.

Two review authors extracted the data from each included report using a standard form created for this review (online supplementary file 2). Discrepancies were discussed until consensus was reached. We contacted authors to obtain additional information when needed.

10.1136/bmjgh-2019-001935.supp2 Supplementary data

### Data analyses

We presented the main characteristics, barriers and enablers of the campaigns descriptively. If data were available, we planned to assess the effectiveness of each campaign by calculating the mean absolute and relative reduction in CS rate and 95% CIs. We planned to combine the results of similar studies and to calculate pooled mean differences or mean standardised differences and 95% CIs. Due to lack of data, this was not possible.

### Patient and public involvement statement

It was not appropriate or possible to involve patients or the public in the design, conduct, reporting or dissemination of our research.

## Results

The electronic search retrieved 14 320 citations. After removing 118 duplicates, 14 202 citations were screened; 14 152 were excluded based on their title and abstract; and 50 were selected for full-text reading. We included 1 study^45^; the other 49 were excluded mainly because they did not report a mass media campaign (online supplementary file 3). Six other reports were identified through contact with experts. The final review included seven reports that described campaigns conducted in Argentina (Cava S, Campaign for the Urgent Reduction of Unnecessary Caesarean Sections), Brazil,^45^ Chile (Sadler M, Campaña #INNEcesareas Chile 2014–2015), Cyprus (Leontiou S, The Normal Birth Campaign 2014–2015: Activities, Challenges, and Opportunities), Iran (Akbari N, Majlesi M, Montazeri A, *et al*, ‘No to Unnecessary Caesarean Section': Evaluation of a Mass Media Campaign on Women's Knowledge, Attitude and Intention for Mode of Delivery, in press), Italy (Montilla P, Merzagora F, Scolaro E, *et al*, Lessons from a Multidisciplinary Partnership Involving Women Parliamentarians to Address the Overuse of Caesarean Section in Italy, in press) and Puerto Rico (Nazario JOM, Campaña #INNEcesareas Puerto Rico 2012–2013) (online supplementary file 4). Six of the included campaigns were not described in printed journal articles; the Cyprus campaign was an oral presentation in a meeting; the campaigns conducted in Argentina, Chile and Puerto Rico were reported in webpages; and two campaigns were manuscripts in press (the Iranian and Italian campaigns). We contacted the authors of all seven reports to obtain additional details. The main characteristics of the campaign are listed in table 1.

10.1136/bmjgh-2019-001935.supp3 Supplementary data

10.1136/bmjgh-2019-001935.supp4 Supplementary data

**Table 1:** Main characteristics of seven mass media campaigns to reduce unnecessary CS

**Characteristic**	**N**	**Country**
**Region**		
South America	3	Argentina, Brazil, Chile
Central America	1	Puerto Rico
Europe	2	Cyprus, Italy
Asia	1	Iran
**Formative research prior to campaign**		
Yes	1	Iran
No	6	Argentina, Brazil, Chile, Cyprus, Italy, Puerto Rico
**Pretesting of messages**		
Yes	1	Iran
No	1	Brazil
Unclear/ no information	5	Argentina, Chile, Cyprus, Italy, Puerto Rico
**Target audience**		
Reproductive age & pregnant women	1	Italy
General public	6	Argentina, Brazil, Chile, Cyprus, Iran, Puerto Rico
**Communication strategies**		
Direct*	2	Argentina, Brazil
Indirect†	7	Argentina, Brazil, Chile, Cyprus, Iran, Italy, Puerto Rico
Both	2	Argentina, Brazil
**Communication channels**		
Written material	3	Cyprus, Italy, Puerto Rico
Radio	1	Argentina
TV (open, paid, or close-circuit)	4	Argentina, Cyprus, Italy, Iran
Internet and social media	5	Argentina, Brazil, Chile, Italy, Puerto Rico
**Celebrities/ famous spokespersons**		
Yes	4	Argentina, Chile, Cyprus, Puerto Rico
No	3	Brazil, Iran, Italy
**Duration of campaign**		
range (min-max)	7 days - 34 months	
less than 1 month	2	Argentina, Iran
1–11 months	2	Chile, Cyprus
12–34 months	2	Brazil, Italy
Unclear/ No information	1	Puerto Rico
**Outcome measured**		
None	5	Argentina, Chile, Cyprus, Italy, Puerto Rico
Knowledge about CS / VD	2	Brazil, Iran
Attitude toward CS /VD	2	Brazil, Iran
Preference for CS / VD	2	Brazil, Iran

*Use of interpersonal contact (face-to-face presentations, performance and group discussions).

†Use of mass media channels (such as TV or newspaper).

CS, caesarean section; TV, television; VD, vaginal delivery.

Details of the objectives and the creation and implementation of the campaigns are presented in tables 2 and 3.

**Table 2:** Main objectives and messages of media campaigns to reduce unnecessary CS

Country	Objectives	Messages
Argentina	Raise awareness about unnecessary CS.	Say ‘no’ to unnecessary CS. Women who deliver vaginally are an endangered species.
Brazil	Increase the public’s knowledge. Promote cultural change to value normal birth. Reduce rates of CS and unnecessary interventions during childbirth.	VD is an important and positive experience for women and children. VD is good for the health of women and children. Think critically about CS rates in Brazil.
Chile	Avoid unnecessary CS. Promote respectful necessary CS. Promote the use of Lamaze recommendations for a physiological birth.	In Chile, there is an excess of CS without any medical indication. Birth is a physiological event that only rarely requires obstetric interventions like a CS. Follow Lamaze recommendations for a physiological birth (eg, wait for spontaneous onset of labour, continuous support during labour and avoid lithotomy position for delivery).
Cyprus	Inform and raise awareness about the mind and body benefits of normal childbirth for families and its impact on society. Strengthen and support couples’ rights to choice in childbirth. Empower midwives and support their role in normal childbirth. Improve national perinatal indicators.	Say ‘yes’ to VD. VD is the best choice for women, babies and families. VD empowers women and midwives. VD leads to stronger families and stronger society and improves health outcomes for all citizens.
Iran	Persuade pregnant women to choose spontaneous VD instead of unnecessary CS.	Give birth naturally to guarantee your own health, that of the baby and of the next generation.
Italy	Raise awareness and foster action of authorities and women to reduce unnecessary CS in the country. Inform women about the indications and risks of CS. Disseminate and promote WHO's policies to achieve optimal CS rates.	A CS is a life-saving surgical procedure that should be used when complications occur. For most women, giving birth should be a natural event. Mode of birth is your choice and should be discussed with your healthcare provider.
Puerto Rico	Prevent and reduce unnecessary CS and other unnecessary interventions during labour, delivery and the postpartum period. Empower Puerto Rican women to face the increasing rates of unnecessary CS as a public health issue. Promote and foster humanised births as the safest and heathiest option for delivery.	You decide, be the protagonist and take charge (of your own birth). CS is a major surgery with risks for mothers and babies. Pregnancy, delivery and the postpartum period are natural events, not diseases that require medical interventions. Humanised childbirth is beneficial for the health of mothers and babies.

CS, caesarean section; VD, vaginal delivery.

**Table 3:** Main characteristics of the creation and implementation of seven mass media campaigns to reduce unnecessary caesarean section

Characteristics	Argentina (2009)	Brazil (2015–2017)	Chile (2014–2015)	Cyprus (2014–2015)	Iran (2016)	Italy (2010–2011)	Puerto Rico (2012–2013)
Main creator	NGO (RELACAHUPAN Argentina)	Minas Gerais Federal University and Belo Horizonte Health Department	NGO (RELACAHUPAN Chile)	Council of Midwives Committee	Multiprofessional expert panel	NGO (ONDa), WHO, National OB-GYN Association and female parlamentarians	Student association, Puerto Rico University Public Health School
Authorities involved in creation/support of campaign	No	Local health department, MoH and local professional association	No	National and local professional association and MoH	No	WHO, National Professional Association and parliamentarians	No
Main funding	Mama Cash (international feminist organisation) and voluntary work (for dissemination)	International, national and local scientific funding agencies, MoH and PAHO	Mostly voluntary work	Mostly voluntary work	Iran University of Medical Science	WHO Partnership for Maternal, Newborn & Child Health	NI
Type of intervention	Part of multicomponent intervention	Part of multicomponent intervention	Isolated intervention	Isolated intervention	Isolated intervention	Part of multicomponent intervention	NI
Design							
Theory used	No	Yes	No	No	Yes	No	NI
Formative research	No	No	No	No	Yes	No	No
Communication experts involved	Yes	Yes	No	Yes	Yes	Yes	NI
Pretesting	No	No	No	No	Yes	No	NI
Target public	General public	General public	General public	General public	General public	Women (pregnant and not pregnant)	General public
Medium							
Type of communication	Direct: group discussions in hospitals/public spaces Indirect: radio and video spots on closed TV in public transportation, internet (YouTube), poster and mailing lists	Direct: itinerant exhibition Indirect: internet channels	Indirect: five video clips posted on Internet channels	Indirect: spot on open TV, posters and pamphlets	Indirect: spot on open TV	Indirect: spot on open and paid TV, internet channels, women’s magazines and pamphlets	Indirect: video, informative posters and pamphlets on internet channels
Celebrities involved	Yes (actress)	No	Yes (actresses)	Yes (singer, actresses and first lady)	No	No	Yes (actors, singers and musicians)
Intensity	NI	NI	NI	NI	Open TV: 10 consecutive days	Paid TV: 3 times/day, 4 days/1 month. Open TV: 3 times/day, 15 days/1 month	NI

MoH, ministry of health; NGO, non-governmental organisation; NI, no information; ONDa, National Observatory for Women's Health; PAHO, Pan American Health Organisation; RELACAHUPAN, Red Latinoamericana y del Caribe para la Humanización del Parto y el Nacimiento; TV, television.

The majority of the campaigns were independent efforts of NGOs, activist organisations or associations strongly engaged in this topic. Only two (Brazil and Cyprus) had the the involvement of the ministry of health, and three (Brazil, Cyprus and Italy) had the involvement of professional associations. Three of the campaigns (Chile, Cyprus and Iran) were stand-alone interventions. Only one of the campaigns (Iran) conducted formative research, and two campaigns (Brazil and Iran) used theories of behavioural change in the development of the campaign. Five of the campaigns (Argentina, Brazil, Cyprus, Iran and Italy) had communication specialists in their creation teams, and four (Argentina, Chile, Cyprus and Puerto Rico) used authority figures/celebrities to disseminate the campaign messages. All campaigns used indirect communication: five disseminated messages through internet channels (Argentina, Brazil, Chile, Italy and Puerto Rico); three used TV spots (Cyprus, Iran and Italy); and four used pamphlets or posters (Argentina, Cyprus, Italy and Puerto Rico). Two of the Latin American campaigns also used direct communication with the target audience. The Argentinean campaign held group discussions with women. The Brazilian campaign^45^ contacted the public through an interactive exhibition that combined different languages (digital art with theatrical techniques) and media (videos, photos, scenarios and panels).

Table 4 presents the main barriers and enablers during the creation and implementation of the campaigns. In both phases, lack of funding, human resources and institutional support was the main barrier, and volunteer work was one of the main enablers.

**Table 4:** Main barriers and enablers of seven mass media campaigns to reduce unnecessary caesarean section

Barriers	Enablers
**Creation of the campaign**	
1. Lack of human resources and time for volunteers to create campaign (Cyprus and Chile).	1. Involvement of communication professionals to design campaign (Brazil).
2. Lack of institutional support (Chile).	2. Support from ministry of health to allow public servants to develop the campaign during working hours (Cyprus).
3. Lack of funding (Chile).	3. Volunteer participation of celebrities in television spots (Cyprus and Argentina).
4. Difficulties of volunteers to organise meetings and plan campaigns (Chile).	4. Volunteer work of professionals in production of good quality videos (Cyprus).
5. Difficulties in finding key persons who would help with creation at no cost (Cyprus).	5. Support of other non-governmental organisations (Argentina).
**Implementation of the campaign**	
1. Lack of funding to transport and display exhibit (Brazil).	1. Trained mediators to help participants get in touch with their senses and emotions (Brazil).
2. Lack of local political support to promote campaign (Brazil).	2. Use of art to affect the sensibility of participants, to touch their hearts and not only their rational side (Brazil).
3. Finding key persons who would help with dissemination without charging (Cyprus).	3. Participation of entertainment celebrities (actresses and singers) in promotional material (Chile and Argentina).
4. Opposition of professional societies, authorities and universities to a campaign that was not created by them (Chile).	4. Avoidance of controversies and dissociation of campaign from extremist/radical groups initiatives or views (Chile and Argentina).
5. Hesitancy of women to accept campaign’s message due to prevailing belief that medicalised births are safer for mother and baby (Chile).	5. Enthusiasm and good will of volunteers in promoting campaign (Chile).
6. Lack of funding for dissemination (Chile) and assessment of outcomes/effects of campaign (Argentina).	6. Good relationships with the key media stakeholders (Italy).
	7. No charge from owners of communication channels to disseminate campaign spots (Argentina).

Table 5 presents the assessment of the effects of the intervention. Five campaigns did not assess any outcome (Argentina, Chile, Cyprus, Italy and Puerto Rico), and the other two (Brazil and Iran) measured changes in knowledge, attitude and preferred route of delivery among the exposed participants using written questionnaires.

**Table 5:** Assessment of effects of seven mass media campaigns to reduce unnecessary CS

Characteristics	Argentina (2009)	Brazil (2015–2017)	Chile (2014–2015)	Cyprus (2014–2015)	Iran (2016)	Italy (2010–2011)	Puerto Rico (2012–2013)
Outcomes assessed	None	Preference for CS. Knowledge of risks/benefits of CS. Opinion on VD.	None	None	Knowledge about childbirth. Attitude towards VD and CS. Intended route of delivery.	None	None
Period of outcome assessment	NA	Immediately after exhibit	NA	NA	10 days after exposure	NA	NA
Sample assessed	NA	Preference for CS: n=1933 general public and n=1287 pregnant women. Knowledge of risks/benefits of CS: n=1933 general public and n=1287 pregnant women. Opinion about VD: n=17 501 visitors.	NA	NA	466 pregnant women (194 had seen the TV spot; 272 had not seen it). All women had no previous CS, were mostly in the second and third trimesters of pregnancy, and attending antenatal care in public and private clinics in Teheran	NA	NA
Tool used to assess effect	NA	Written questionnaires immediately before and after the exhibit			Written questionnaires before and after TV campaign		
Effects	NA	Decrease in preference for CS (14.7%×10.4%, p=0.006). Increase in good/very good knowledge about CS risks (50.5%×71.5%, p<0.001). Decrease in opinion that VD was very bad or bad (12.2%×1.9%, p<0.001).	NA	NA	Changes in 194 women exposed to campaign: Increase in knowledge scores (p=0.008). Increase in attitude scores towards VD (p=0.05). Decrease in intention to deliver by CS (39.2%×24.7%, p=0.004). No changes in attitude scores towards CS.	NA	NA

CS, caesarean section; NA, not applicable; TV, television; VD, vaginal delivery.

Due to lack of data, we could not pool the results from individual studies into a combined estimate to assess the effectiveness of the campaigns. We therefore present a narrative description of the most important characteristics and findings of each campaign, as well as the barriers and facilitators reported by the authors.

### Argentina: ‘Campaign for the Urgent Reduction of Unnecessary Caesarean Sections’

There is no publication of this campaign. We obtained information through its website and interviews with the main coordinator. The campaign occurred in May 2009, during the ‘International Week for Respecting Childbirth’. It was created by the Argentinean chapter of the international NGO network Red Latinoamericana y del Caribe para la Humanización del Parto y el Nacimiento (RELACAHUPAN) as part of a multicomponent intervention that took place during the week in the country. RELACAHUPAN is a Latin American network of NGOs that spans 20 countries.^46^ The goals of this network are to improve the experience of birth, to promote humanised births, and to guarantee the rights of women to make informed decisions about pregnancy and childbirth, emphasising the benefits of natural birth. Volunteers from the NGO promoted group discussions about the messages of the campaign with women in the waiting areas of hospitals and public places, with poster displays. A short video featuring a famous TV and film actress (who worked pro bono) was created and broadcast on closed-circuit TV in public transportation and on the internet. The video had close-caption legends in several languages besides Spanish. Mama Cash, an international feminist association, provided funding to RELACAHUPAN, which was used in part for the creation of this video. There is no information on the estimated cost of the campaign nor on the intensity of messaging, nor the number of persons exposed to the campaign. No outcomes were measured.

### Brazil: ‘The Senses of Birth Campaign’

The campaign occurred between 2015 and 2017 (34 months) in five cities in Brazil. It was created by health professionals and involved experts in communication and museum exhibits. The campaign was part of a long-term multisectorial initiative led by the municipal health department to increase VD in the city of Belo Horizonte (‘BH pelo Parto Normal’), which started in 2007 and lasted 10 years. The campaign consisted of an interactive exhibition that combined digital art with theatrical techniques, videos, photos, scenarios and panels to promote an emotional experience, engage the visitors and encourage them to think critically. The free exhibition consisted of a 40 min guided interactive circuit that took place inside five containers parked on outdoor spaces in different locations (near shopping centres, in public parks, schools and universities). Immediately after going through the experience, visitors were invited to participate in group chats. This itinerant exhibit remained in each site for several weeks and was then packed up and transported to other locations by truck. The campaign also involved indirect communication through a website, Facebook, Instagram pages and a YouTube channel. The campaign cost approximately US$350 000 per year to cover 12 exhibits in the five cities. It was funded by national scientific agencies, the Brazilian Ministry of Health, the Bill and Melinda Gates Foundation and the Pan-American Health Organisation.^45^

A total of 42 170 persons of all ages visited the exhibits in the five cities. There were 8204 followers on Facebook, 4505 on Instagram and 2900 on YouTube. The authors used written questionnaires to measure changes in visitors’ preferences for route of delivery, knowledge about risks of CS and opinion on VD. There were significant changes in these three measures (table 5). The investigators did not assess changes in the rates of CS as one of their outcomes.

### Chile: ‘InneCesareas’

There are no publications of this campaign. Data were obtained through interviews with one of its creators, who also provided the web content of the campaign. This national, internet-based campaign lasted 6 months (November 2013–May 2014). It was created by the Chilean chapter of the international NGO network RELACAHUPAN.^46^ The campaign was a stand-alone intervention created by volunteers from RELACAHUPAN Chile. The messages were delivered through five short videos posted on YouTube, Facebook and Twitter. Famous soap opera actresses participated in the videos, as well as local authority figures (famous obstetricians and midwives), at no cost. A journalist produced the videos. The creators of the campaign promoted the initiative by appearing in talk shows and interviews. The campaign cost US$400 for the production of the videos. This was obtained through fundraising activities among RELACAHUPAN Chile members. There were approximately 50 000 visits to the YouTube videos. The effectiveness of the campaign was not assessed.

### Cyprus: ‘Normal Birth Campaign’

This report was presented only as an oral communication in an international meeting. Additional details were obtained by contacting the presenter. This national campaign took place in Cyprus between 2014 and 2015 (12 months). It was designed as a stand-alone intervention by the Council of Midwives Committee and the Ministry of Health. Communication experts from TV channels volunteered to help create the campaign’s video clip. The campaign was supported by the country’s first lady, national specialist societies, religious authorities, several NGOs and civil trade unions. The messages were delivered through indirect communication, including posters, banners and pamphlets in local hospitals, and press releases that were sent through emails and mass media posts. The central piece of the campaign was a short video clip, with local female celebrities, that was disseminated in local open TV channels during 12 months. There is no information on the frequency of the campaign messages. The cost of the campaign was approximately €5000, and it was funded by the Cyprus Nurses and Midwives Association, trade unions and the Nursing and Midwives Council. There is no information on the estimated number of persons exposed to the campaign. No outcomes were measured or reported. The effectiveness of the campaign was not assessed.

### Iran: ‘No to Unnecessary Caesarean Section’

This prepublication manuscript (obtained from the authors) describes a national campaign conducted in Iran during 10 days in April 2016. The campaign was created by an expert panel of nine persons (academic obstetricians and midwives, health education experts and advertising professionals). There is no information on the cost of the campaign. The campaign consisted of a short video clip that was broadcast on four of the country’s eight open TV channels during 10 consecutive days and in antenatal care clinics (on closed-circuit TV). There is no information on the estimated number of persons exposed to the TV campaign. The authors measured changes in knowledge, attitude and preferred route of delivery of 466 pregnant women without a previous CS and living in Teheran for at least 6 months, who were managed in public and private antenatal care clinics in that city. The authors used written questionnaires to assess all participants before and after the campaign. Most of the participants (58%, n=272) stated they had not seen the TV spots when they answered the second questionnaire. The 194 exposed women had a significant increase in knowledge and attitude scores towards VD and a significant decrease in the proportion of pregnant women who intended to have a CS, but no significant changes in attitude towards CS (table 5).

### Italy: ‘Caesarean Section: When and Why’

A manuscript describing this campaign is in press and was shared by the authors. Additional information was obtained by contacting representatives of various partners involved in this initiative. The nationwide campaign took place between January 2010 and September 2011 (21 months). The campaign was created by ONDa (National Observatory for Women's Health), an NGO focused on women’s health, with the participation of the World Health Organization (WHO), the Italian Society of Gynaecologists and Obstetricians and a group of Italian female bipartisan parliamentarians. The campaign was part of a multicomponent intervention that included technical meetings at WHO to increase the awareness of Italian parliamentarians about the overuse of CS and to foster political action at national and regional levels to promote vaginal births and to reduce CS. The main elements of this campaign were a TV spot (created pro bono by a professional Italian marketing and advertising company), an online survey disseminated by a national women’s magazine and an educational brochure about indications and risks of CS. A short TV spot was initially broadcast on open and paid TV channels three to four times/day for up to 2 weeks and then posted on YouTube and campaign websites. The largest Italian women’s magazine featured a text about CS in Italy and invited readers to answer an online anonymous survey about their preferences for and knowledge about the risks of CS versus VD. The magazine also distributed a special brochure with information on CS, including indications and risks. This brochure was also available in hospitals and clinics. There is no information on the estimated number of persons exposed to the campaign. No outcomes were measured, and the effectiveness of the campaign was not assessed.

### Puerto Rico: ‘INNEcesareas’

There are no publications of this campaign. Data were obtained through personal contact with the campaign coordinator. This national campaign was created by the Maternal and Child Health Student Association of Puerto Rico’s University School of Public Health. The messages were delivered using indirect communication through a hip-hop video posted on the campaign webpage and internet channels (YouTube, Facebook and Twitter). Celebrities (actors, musicians and other artists) acted as spokespersons pro bono. No outcomes were measured, and there is no information on the number of persons exposed to the campaign or its effectiveness.

## Discussion

This review identified seven mass media campaigns directed at lay people with the specific objective of reducing unnecessary CS, only one of which was published in a scientific journal. In their creation phase, only one of the campaigns used formative research to inform the design and implementation of the intervention and obtained input from the target population (through pilot testing) before being launched. The involvement of health authorities was not frequent. None of the campaigns assessed its effects on CS rates. Only two campaigns measured their impacts on participants’ knowledge, attitudes and birth preferences, but only in the short term. The main barriers to the creation and implementation of the campaigns were lack of financial and human resources. The main enablers were the enthusiasm of the volunteers, the participation of famous persons/celebrities and the involvement of communication professionals.

The lack of formative research and testing in six of the seven campaigns was surprising. Formative research is essential to assess knowledge, awareness and beliefs of the population, to understand local cultural norms, to ensure that the content and format of the campaign are appropriate and acceptable, and to identify the most credible and most persuasive channels and spokespersons.^29 30 41–43 47^

Several campaigns depicted messages about the value of VD. Overall, in the material produced with the aim of reducing CS, the benefits of VD are often lost in the midst of messages focused on the potential adverse consequences of CS. We believe this perspective is important and should be included in future campaigns since women welcome this information and value what is best for their babies and themselves.^48^

The specific types of communication used by the campaigns varied, but they were mostly indirect, that is, did not involve personal contact with the target audience. Traditionally, most mass media campaigns rely on the use of posters, handouts, public service announcements and presentations, along with information disseminated in newspapers, magazines, radio and TV.^47^ Three of the campaigns included in this review (Chile, Iran and Italy) created a short film that was produced pro bono and broadcast on open TV. The costs involved in using TV can be a barrier, and funding should be secured. In recent years, with the availability and popularisation of new media technologies, mass media campaign dissemination has expanded via websites, social networking, smart phone apps and online messaging platforms.^49 50^ Studies on the effectiveness of these new media health campaigns report mixed results.^51–54^ Most of the campaigns included in our review used internet media, and the two that measured the number of accesses (Brazil and Chile) reported a high number of visitors to their sites, which is an indication of the potential outreach of and interest in these campaigns.

We could not assess the effectiveness of the campaigns in reducing the rates of CS because none of the included studies measured this outcome. Soft outcomes (knowledge, attitude, behaviour or preferences) were assessed in two campaigns (Brazil and Iran) but only immediately after exposure to the campaign. The duration of mass media effects, that is, the persistence of the positive effects of mass media on knowledge, attitude, behaviour or preferences over time, is considered an important element in assessing the effectiveness of mass media interventions.^27 28 44^ This was not reported by any of the campaigns. The lack of an evaluation component of campaigns is not uncommon. In a systematic review on campaigns about child survival, Naugle and Hornik reported that 54% of over 100 studies did not measure and report any behavioural or health outcome.^27^

Our review has several strong points. To our knowledge, this is the first review on this topic. We designed a broad and sensitive search strategy and used rigorous methodology to reduce bias. Extensive efforts were undertaken to contact experts in the field to identify campaigns not identified through electronic searches. Six of the seven included campaigns were uncovered using this strategy. We acknowledge that, despite our extensive search, we may have missed relevant reports. An additional limitation of this review is that many reports were incomplete and did not provide all relevant details, which can be an obstacle to the replication of similar campaigns in other settings. Due to the lack of details and outcome assessments, we are not able to infer what elements of a mass media campaign on this topic could be most associated with success.

Mass media campaigns have been recommended as a key component of comprehensive strategies aiming at behavioural change.^47^ However, our findings indicate that most of the campaigns to reduce unnecessary CS are almost ‘amateur’ efforts relying on volunteers, and counting on people’s good will and pro bono work to support their activities, without rigorous evaluation to assess whether the campaigns actually achieved their aim or not. There is a clear need for more, adequately designed and reported studies in this area, including research on cost-effectiveness.

Importantly, the success of any campaign to reduce unnecessary CS will depend on the existence of facilities that offer women safe and adequately resourced environments to encourage and support them during labour and vaginal birth. Suboptimal quality of care, disrespect and abuse, insufficient communication or perverse relationships between women and providers must be addressed for women to prefer VD.^48 55^ Successful mass media campaigns targeted at the general public can play an important role in raising awareness. However, high CS rates are not due exclusively to the preferences of women. Therefore, initiatives to reduce CS need to involve and target the other contributors to this phenomenon, such as healthcare providers, hospitals, policy makers and organisations responsible for paying for medical procedures.^37 55^

## Conclusions

This review found seven mass media campaigns directed at laypersons to reduce CS, only one of which was published. Most campaigns did not use key principles recommended for the creation and implementation of health communication interventions, and none assessed the effects of the campaigns in reducing CS rates. There is a need for more well-designed and well-reported studies in this area, including rigorous research to assess impact.
