# Supplementary material for: Mass media campaigns to reduce unnecessary caesarean sections: a systematic review
Source: BMJ Glob Health. 2020 Feb 26;5(2):e001935. doi: 10.1136/bmjgh-2019-001935 (PMC7146028; doi:10.1136/bmjgh-2019-001935)
Supplement: Supplementary data [file bmjgh-2019-001935supp001.pdf]

Supplementary file 1. Search strategies for SR on campaigns to reduce caesarean sections

PUBMED DATABASE<br>http://www.pubmed.gov	
**Concept**	**Search string**
#1	"Health Education"[Mesh] OR "health education"[TW] OR "promotion"[TW] OR Health Literacy[Mesh] OR mhealth[TW] OR "government publications as topic"[MESH] OR "information dissemination"[MESH] OR "Health promotion"[MESH] OR "mass media"[MeSH] OR "mass medium" [TW] OR "mass media" [TW] OR "mass communication"[TW] OR "mass information"[TW] OR campaign*[TW] OR advert*[TW] OR "social marketing"[MESH] OR "social marketing"[TW] OR broadcast* [TW] OR pamphlets [Mesh] OR pamphlet*[TW] OR serial publication*[TW] OR brochure*[TW] OR leaflet*[TW] OR edutainment[TW] OR infotainment[TW] OR "advertising as topic"[MESH] OR "persuasive communication"[MESH] OR message* [TW] OR marketing [TW] OR publicity[TW] OR public information[TW] OR positive framing[TW] OR poster[TW] OR posters[TW] OR billboard*[TW] OR visual art*[TW] OR street art*[TW] OR "famous persons"[MESH] OR spokesperson*[TW] OR celebrit*[TW] OR public relation*[TW] OR "Communications Media"[Mesh] OR Mass Media[TW] OR Mass Medium[TW] OR Internet[Mesh] OR internet [TW] OR "social media"[TW] OR radio[TW] OR television [TW] OR TV[TI] OR Newspapers [TW] OR "tape recording"[MESH] OR Videotape[TW] OR "Motion Pictures" [TW] OR Telecommunication[TW] OR "video recording"[MESH] OR Videorecording[TW] OR "Audiovisual Aids" [TW] OR "audiovisual equipment" [TW] OR facebook [TW] OR twitter [TW] OR youtube [TW] OR ICT[TIAB] OR "soap opera" [TW] OR blog* [TW] OR instagram [TW] OR cinema*[TW] OR movie*[TW] OR film*[TW] OR magazin*[TW] OR magasin* [TW] OR phone*[TW] OR telephone*[TW] OR electronic mail*[TW] OR email*[TW] OR e-mail*[TW] OR webmail*[TW] OR mailing list*[TW] OR discussion list*[TW] OR listserv* [TW] OR phone* [TW] OR telephone*[TW] OR text [TI] OR texting [TW] OR texted [TW] OR sms [TW] OR mms [TW] OR audio*[TW] OR video[TW] OR videos[TW] OR cassette*[TW] OR tape*[TW] OR dvd*[TW] OR compact dis*[TW] OR cd [TW] OR cds[TW] OR multimedia[TW] OR multi media [TW] OR web[TW] OR website*[TW] OR online[TW] OR weblog*[TW] OR podcast*[TW] OR portal*[TW] OR e-communication*[TW] OR electronic communication*[TW] OR computer program*[TW] OR computer mediated[TW] OR apps[TW] OR tweet[TW] OR bebo[TW] OR myspace[TW] OR chatroom[TW] OR chatrooms[TW] OR viral message[TW] OR wiki*[TW] OR virtual*[TW] OR "books"[MESH] OR booklet*[TW] OR flyer*[TW] OR Printed media[TW] OR print media[TW] OR printed material*[TW] OR print material*[TW] OR publication*[TW] OR "publications"[MESH] OR festival*[TW] OR entertainment [TW] OR (("community intervention" [TW] OR "Community Networks"[Mesh] OR "Community Health Planning"[Mesh]) AND Media [TW]) OR (information [TW] AND (distribute[TW] OR distributed[TW] OR distribution[TW] OR disseminat*[TW])) OR ((raise[TW] OR raised[TW] OR raising[TW]) AND awareness) OR ((community[TW] OR broadbased[TW] OR broad based[TW] OR public[TW]) AND education program*[TW]) OR (promotion*[TW] AND (item[TW] OR items[TW] OR material[TW] OR materials [TW])) OR (communication [TW] AND (program*[TW] OR strategy[TW] OR strategies[TW])) OR (famous[TW] AND (person[TW] OR persons[TW] OR person's[TW] OR people[TW])) OR ((virtual[TW] OR indirect[TW] OR record[TW] OR records[TW] OR recording*[TW] OR film[TW] OR films[TW] OR filmed[TW] OR audio*[TW]) AND (social contact[TW] OR testimon*[TW] OR story[TW] OR story's[TW] OR stories[TW] OR account[TW] OR experience*[TW] OR narrative*[TW] OR play[TW] OR ethnograph*[TW]))
#2	"Natural Childbirth"[Mesh] OR "normal childbirth" OR "normal birth" OR "vaginal birth" OR "vaginal childbirth" OR "Cesarean Section"[Mesh] OR "Abdominal Deliveries"[TW] OR "C Section" [TW] OR "C

	Sections" [TW] OR Postcesarean[TW] OR Postcaesarean[TW] OR Cesarean[TW] OR Caesarean [TW] OR "Vaginal Birth after Cesarean"[Mesh]
#3	#1 AND #2

EMBASE DATABASE<br>http://www.embase.com	
Concept	Search string
#1	('childbirth education'/exp OR "health education":ti,ab,de,tn OR promotion:ti,ab,de,tn OR 'Health Literacy'/exp OR mhealth:ti,ab,de,tn OR 'government publication'/exp OR 'information dissemination'/exp OR 'Health promotion'/exp OR 'mass communication'/exp OR "mass medium":ti,ab,de,tn OR "mass media":ti,ab,de,tn OR "mass communication":ti,ab,de,tn OR "mass information":ti,ab,de,tn OR 'medical information'/exp OR 'information service'/exp OR 'information literacy'/exp OR 'patient information'/exp OR 'therapeutic misconception'/exp OR 'medical illustration'/exp OR campaign*:ti,ab,de,tn OR advert*:ti,ab,de,tn OR 'social marketing'/exp OR "social marketing":ti,ab,de,tn OR broadcast*:ti,ab,de,tn OR 'publication'/exp OR pamphlet*:ti,ab,de,tn OR "serial publication*":ti,ab,de,tn OR brochure*:ti,ab,de,tn OR leaflet*:ti,ab,de,tn OR edutainment:ti,ab,de,tn OR infotainment:ti,ab,de,tn OR 'advertising'/exp OR 'persuasive communication'/exp OR message*:ti,ab,de,tn OR marketing:ti,ab,de,tn OR publicity:ti,ab,de,tn OR "public information":ti,ab,de,tn OR "positive framing":ti,ab,de,tn OR poster:ti,ab,de,tn OR posters:ti,ab,de,tn OR billboard*:ti,ab,de,tn OR "visual art*":ti,ab,de,tn OR "street art*":ti,ab,de,tn OR 'public figure'/exp OR spokesperson*:ti,ab,de,tn OR celebrit*:ti,ab,de,tn OR "public relation*":ti,ab,de,tn OR "Mass Media":ti,ab,de,tn OR "Mass Medium":ti,ab,de,tn OR 'Internet'/exp OR internet:ti,ab,de,tn OR "social media":ti,ab,de,tn OR radio:ti,ab,de,tn OR television:ti,ab,de,tn OR TV:ti OR Newspapers:ti,ab,de,tn OR 'recording'/exp OR Videotape:ti,ab,de,tn OR "Motion Pictures":ti,ab,de,tn OR Telecommunication:ti,ab,de,tn OR Videorecording:ti,ab,de,tn OR "Audiovisual Aids":ti,ab,de,tn OR "audiovisual equipment":ti,ab,de,tn OR facebook:ti,ab,de,tn OR twitter:ti,ab,de,tn OR youtube:ti,ab,de,tn OR ICT:ti,ab OR "soap opera":ti,ab,de,tn OR blog*:ti,ab,de,tn OR instagram:ti,ab,de,tn OR cinema*:ti,ab,de,tn OR movie*:ti,ab,de,tn OR film*:ti,ab,de,tn OR magazin*:ti,ab,de,tn OR magasin*:ti,ab,de,tn OR phone*:ti,ab,de,tn OR telephone*:ti,ab,de,tn OR "electronic mail*":ti,ab,de,tn OR email*:ti,ab,de,tn OR e-mail*:ti,ab,de,tn OR webmail*:ti,ab,de,tn OR "mailing list*":ti,ab,de,tn OR "discussion list*":ti,ab,de,tn OR listserv*:ti,ab,de,tn OR phone*:ti,ab,de,tn OR telephone*:ti,ab,de,tn OR text:ti OR texting:ti,ab,de,tn OR texted:ti,ab,de,tn OR sms:ti,ab,de,tn OR mms:ti,ab,de,tn OR audio*:ti,ab,de,tn OR video:ti,ab,de,tn OR videos:ti,ab,de,tn OR cassette*:ti,ab,de,tn OR tape*:ti,ab,de,tn OR dvd*:ti,ab,de,tn OR "compact dis*":ti,ab,de,tn OR cd:ti,ab,de,tn OR cds:ti,ab,de,tn OR multimedia:ti,ab,de,tn OR "multi media":ti,ab,de,tn OR web:ti,ab,de,tn OR website*:ti,ab,de,tn OR online:ti,ab,de,tn OR weblog*:ti,ab,de,tn OR podcast*:ti,ab,de,tn OR portal*:ti,ab,de,tn OR e-communication*:ti,ab,de,tn OR "electronic communication*":ti,ab,de,tn OR "computer program*":ti,ab,de,tn OR "computer mediated":ti,ab,de,tn OR apps:ti,ab,de,tn OR tweet:ti,ab,de,tn OR bebo:ti,ab,de,tn OR myspace:ti,ab,de,tn OR chatroom:ti,ab,de,tn OR chatrooms:ti,ab,de,tn OR "viral message":ti,ab,de,tn OR wiki*:ti,ab,de,tn OR virtual*:ti,ab,de,tn OR 'book'/exp OR booklet*:ti,ab,de,tn OR flyer*:ti,ab,de,tn OR "Printed media":ti,ab,de,tn OR "print media":ti,ab,de,tn OR "printed material*":ti,ab,de,tn OR "print material*":ti,ab,de,tn OR publication*:ti,ab,de,tn OR 'publications'/exp OR festival*:ti,ab,de,tn OR entertainment:ti,ab,de,tn) OR (("community intervention":ti,ab,de,tn OR 'community health nursing'/exp OR 'community program'/exp) AND Media:ti,ab,de,tn) OR (information:ti,ab,de,tn AND (distribute:ti,ab,de,tn OR distributed:ti,ab,de,tn OR distribution:ti,ab,de,tn OR disseminat*:ti,ab,de,tn)) OR ((raise:ti,ab,de,tn OR raised:ti,ab,de,tn OR raising:ti,ab,de,tn) AND awareness) OR ((community:ti,ab,de,tn OR

	broadbased:ti,ab,de,tn OR "broad based":ti,ab,de,tn OR public:ti,ab,de,tn) AND "education program*":ti,ab,de,tn) OR (promotion*:ti,ab,de,tn AND (item:ti,ab,de,tn OR items:ti,ab,de,tn OR material:ti,ab,de,tn OR materials:ti,ab,de,tn)) OR (communication:ti,ab,de,tn AND (program*:ti,ab,de,tn OR strategy:ti,ab,de,tn OR strategies:ti,ab,de,tn)) OR (famous:ti,ab,de,tn AND (person:ti,ab,de,tn OR persons:ti,ab,de,tn OR people:ti,ab,de,tn)) OR ((virtual:ti,ab,de,tn OR indirect:ti,ab,de,tn OR record:ti,ab,de,tn OR records:ti,ab,de,tn OR recording*:ti,ab,de,tn OR film:ti,ab,de,tn OR films:ti,ab,de,tn OR filmed:ti,ab,de,tn OR audio*:ti,ab,de,tn) AND ("social contact":ti,ab,de,tn OR testimon*:ti,ab,de,tn OR story:ti,ab,de,tn OR storys:ti,ab,de,tn OR stories:ti,ab,de,tn OR account:ti,ab,de,tn OR experience*:ti,ab,de,tn OR narrative*:ti,ab,de,tn OR play:ti,ab,de,tn OR ethnograph*:ti,ab,de,tn))
#2	('Natural Childbirth'/exp OR 'vaginal delivery'/exp OR "natural childbirth" OR "normal childbirth" OR "normal birth" OR "vaginal birth" OR "vaginal childbirth" OR "vaginal delivery" OR 'Cesarean Section'/exp OR "Abdominal Deliveries":ti,ab,de,tn OR "C Section":ti,ab,de,tn OR "C Sections":ti,ab,de,tn OR Postcesarean:ti,ab,de,tn OR Postcaesarean:ti,ab,de,tn OR Cesarean:ti,ab,de,tn OR Caesarean:ti,ab,de,tn OR 'Vaginal Birth after Cesarean'/exp)
#3	#1 AND #2

Cinahl DATABASE<br>EBSCO http://www.ebsco.com	
Concept	Search string
#1	((MH "Health Education+") OR "health education" OR promotion OR (MH "Health Literacy+") OR mhealth OR (MH "Governement Publications") OR (MH "Health Informtion Networks") OR (MH "Community Health Services") OR (MH "Community Networks") OR (MH "Health promotion+") OR (MH "Communications Media+") OR "mass medium" OR "mass media" OR "mass communication" OR "mass information" OR campaign* OR advert* OR (MH "social marketing+") OR "social marketing" OR broadcast* OR (MH "Pamphlets") OR pamphlet* OR "serial publication*" OR brochure* OR leaflet* OR edutainment OR infotainment OR (MH "advertising") OR (MH "persuasive communication") OR message* OR marketing OR publicity OR "public information" OR "positive framing" OR poster OR posters OR billboard* OR "visual art*" OR "street art*" OR (MH "public figures") OR spokesperson* OR celebrit* OR "public relation*" OR (MH "Communications Media+") OR "Mass Media" OR "Mass Medium" OR (MH "Internet") OR internet OR "social media" OR radio OR television OR TI TV OR Newspapers OR (MH "audiorecording") OR Videotape OR "Motion Pictures" OR Telecommunication OR (MH "videorecording") OR Videorecording OR "Audiovisual Aids" OR "audiovisual equipment" OR facebook OR twitter OR youtube OR TI ICT OR AB ICT OR "soap opera" OR blog* OR instagram OR cinema* OR movie* OR film* OR magazin* OR magasin* OR phone* OR telephone* OR "electronic mail*" OR email* OR e-mail* OR webmail* OR "mailing list*" OR "discussion list*" OR listserv* OR phone* OR telephone* OR TI text OR texting OR texted OR sms OR mms OR audio* OR video OR videos OR cassette* OR tape* OR dvd* OR "compact dis*" OR cd OR cds OR multimedia OR "multi media" OR web OR website* OR online OR weblog* OR podcast* OR portal* OR e-communication* OR "electronic communication*" OR "computer program*" OR "computer mediated" OR apps OR tweet OR bebo OR myspace OR chatroom OR chatrooms OR "viral message" OR wiki* OR virtual* OR (MH "books") OR booklet* OR flyer* OR "Printed media" OR "print media" OR "printed material*" OR "print material*" OR publication* OR (MH "publications+") OR festival* OR entertainment) OR (("community intervention" OR (MH "Community Networks") OR (MH "Community Health Services")) AND Media) OR (information AND (distribute OR distributed OR distribution OR disseminat*)) OR ((raise OR raised OR raising) AND awareness) OR ((community OR broadbased OR "broad based" OR public) AND "education program*") OR (promotion* AND (item OR items OR material OR materials)) OR (communication AND (program* OR strategy OR strategies))

	OR (famous AND (person OR persons OR person's OR people)) OR ((virtual OR indirect OR record OR records OR recording* OR film OR films OR filmed OR audio*) AND ("social contact" OR testimon* OR story OR story's OR stories OR account OR experience* OR narrative* OR play OR ethnograph*))
#2	(MH "Prepared Childbirth") OR "normal childbirth" OR "normal birth" OR "vaginal birth" OR "vaginal childbirth" OR (MH "Cesarean Section") OR "Abdominal Deliveries" OR "C Section" OR "C Sections" OR Postcesarean OR Postcaesarean OR Cesarean OR Caesarean OR (MH "Vaginal Birth after Cesarean")
#3	#1 AND #2

PyscInfo DATABASE<br>EBSCO http://www.ebsco.com	
Concept	Search string
#1	((DE "Health Education+") OR "health education" OR promotion OR (DE "Health Literacy") OR mhealth OR (DE "Community Health") OR (DE "Health promotion") OR (DE "Mass Media") OR "mass medium" OR "mass media" OR "mass communication" OR "mass information" OR campaign* OR advert* OR (DE "social marketing") OR "social marketing" OR broadcast* OR (DE "Reading Materials") OR pamphlet* OR "serial publication*" OR brochure* OR leaflet* OR edutainment OR infotainment OR (DE "advertising") OR (DE "television Advertising") OR (DE "persuasive communication") OR message* OR marketing OR publicity OR "public information" OR "positive framing" OR poster OR posters OR billboard* OR "visual art*" OR "street art*" OR spokesperson* OR celebrit* OR "public relation*" OR (DE "Communications Media") OR "Mass Media" OR "Mass Medium" OR (DE "Internet") OR (DE "Computer Mediated Communication") OR (DE "Electronic Communication") OR internet OR "social media" OR radio OR television OR TI TV OR Newspapers OR (DE "Audiovisual Communications Media") OR Videotape OR "Motion Pictures" OR Telecommunication OR (DE "videorecording") OR Videorecording OR "Audiovisual Aids" OR "audiovisual equipment" OR facebook OR twitter OR youtube OR TI ICT OR AB ICT OR "soap opera" OR blog* OR instagram OR cinema* OR movie* OR film* OR magazin* OR magasin* OR phone* OR telephone* OR "electronic mail*" OR email* OR e-mail* OR webmail* OR "mailing list*" OR "discussion list*" OR listserv* OR phone* OR telephone* OR TI text OR texting OR texted OR sms OR mms OR audio* OR video OR videos OR cassette* OR tape* OR dvd* OR "compact dis*" OR cd OR cds OR multimedia OR "multi media" OR web OR website* OR online OR weblog* OR podcast* OR portal* OR e-communication* OR "electronic communication*" OR "computer program*" OR "computer mediated" OR apps OR tweet OR bebo OR myspace OR chatroom OR chatrooms OR "viral message" OR wiki* OR virtual* OR (DE "books") OR booklet* OR flyer* OR "Printed media" OR "print media" OR "printed material*" OR "print material*" OR publication* OR (DE "books") OR festival* OR entertainment OR (DE "Telecommunications Media") OR (DE "Multimedia") OR (DE "Social Media")) OR<br>(("community intervention" OR (DE "Community Networks") OR (DE "Community Health Services")) AND Media) OR (information AND (distribute OR distributed OR distribution OR disseminat*)) OR ((raise OR raised OR raising) AND awareness) OR ((community OR broadbased OR "broad based" OR public) AND "education program*") OR (promotion* AND (item OR items OR material OR materials)) OR (communication AND (program* OR strategy OR strategies)) OR (famous AND (person OR persons OR person's OR people)) OR ((virtual OR indirect OR record OR records OR recording* OR film OR films OR filmed OR audio*) AND ("social contact" OR testimon* OR story OR story's OR stories OR account OR experience* OR narrative* OR play OR ethnograph*))
#2	(DE "Natural Childbirth") OR "normal childbirth" OR "normal birth" OR "vaginal birth" OR "vaginal childbirth" OR (DE "Cesarean Birth") OR "Abdominal Deliveries" OR "C Section" OR "C Sections" OR Postcesarean OR Postcaesarean OR Cesarean OR Caesarean

#3	#1 AND #2

Web of Science Core Collection DATABASE<br>www.webofknowledge.com/	
Concept	Search string
#1	(("health education" OR promotion OR "Health Literacy" OR mhealth OR "government publications as topic" OR "information dissemination" OR "Health promotion" OR "mass media" OR "mass medium" OR "mass communication" OR "mass information" OR campaign* OR advert* OR "social marketing" OR broadcast* OR pamphlet* OR "serial publication*" OR brochure* OR leaflet* OR edutainment OR infotainment OR "advertising" OR "persuasive communication" OR message* OR marketing OR publicity OR "public information" OR "positive framing" OR poster OR posters OR billboard* OR "visual art*" OR "street art*" OR "famous persons" OR spokesperson* OR celebrit* OR "public relation*" OR "Communications Media" OR "Mass Media" OR "Mass Medium" OR Internet OR internet OR "social media" OR radio OR television OR TV OR Newspapers OR "tape recording" OR Videotape OR "Motion Pictures" OR Telecommunication OR "video recording" OR Videorecording OR "Audiovisual Aids" OR "audiovisual equipment" OR facebook OR twitter OR youtube OR ICT OR "soap opera" OR blog* OR instagram OR cinema* OR movie* OR film* OR magazin* OR magasin* OR phone* OR telephone* OR "electronic mail*" OR email* OR e-mail* OR webmail* OR "mailing list*" OR "discussion list*" OR listserv* OR phone* OR telephone* OR text OR texting OR texted OR sms OR mms OR audio* OR video OR videos OR cassette* OR tape* OR dvd* OR "compact dis*" OR cd OR cds OR multimedia OR "multi media" OR web OR website* OR online OR weblog* OR podcast* OR portal* OR e-communication* OR "electronic communication*" OR "computer program*" OR "computer mediated" OR apps OR tweet OR bebo OR myspace OR chatroom OR chatrooms OR "viral message" OR wiki* OR virtual* OR books OR booklet* OR flyer* OR "Printed media" OR "print media" OR "printed material*" OR "print material*" OR publication* OR publications OR festival* OR entertainment) OR (("community intervention" OR ("Community Networks") OR ("Community Health Services")) AND Media) OR (information AND (distribute OR distributed OR distribution OR disseminat*)) OR ((raise OR raised OR raising) AND awareness) OR ((community OR broadbased OR "broad based" OR public) AND "education program*") OR (promotion* AND (item OR items OR material OR materials)) OR (communication AND (program* OR strategy OR strategies)) OR (famous AND (person OR persons OR person's OR people)) OR ((virtual OR indirect OR record OR records OR recording* OR film OR films OR filmed OR audio*) AND ("social contact" OR testimon* OR story OR story's OR stories OR account OR experience* OR narrative* OR play OR ethnograph*)))
#2	("Natural Childbirth" OR "normal childbirth" OR "normal birth" OR "vaginal birth" OR "vaginal childbirth" OR "Cesarean Section" OR "Abdominal Deliveries" OR "C Section" OR "C Sections" OR Postcesarean OR Postcaesarean OR Cesarean OR Caesarean)
#3	#1 AND #2

Popline DATABASE (Using “POPLINE Advanced Search” tab)	
Concept	Search string
#1	HEALTH EDUCATION OR EDUCATIONAL METHODS OR GOVERNMENT PUBLICATION OR INFORMATION DISSEMINATION OR PROMOTION OR MASS MEDIA OR SOCIAL MARKETING OR ADVERTISING OR BROADCAST MEDIA OR INFORMATION COMMUNICATION TECHNOLOGY OR FILM AND VIDEO OR COMMUNICATION PROGRAMS OR CAMPAIGNS OR PROPAGANDA OR AUDIOVISUAL AIDS OR RADIO OR TELEVISION OR BEHAVIOR CHANGE COMMUNICATION OR INFORMATION NETWORKS
#2	Cesarean Section OR "normal childbirth" OR "normal birth" OR "vaginal birth" OR "vaginal childbirth" OR "Abdominal Deliveries" OR "C Section" OR "C Sections" OR Postcesarean OR Postcaesarean OR Cesarean OR Caesarean
#3	#1 AND #2

EBSCO – MultiDatabase Search http://www.ebsco.com	
Concept	Search string
#1	TI ("health education" OR "promotion" OR mhealth OR "mass medium" OR "mass media" OR "mass communication" OR "mass information" OR campaign* OR advert* OR "social marketing" OR broadcast* OR pamphlet* OR "serial publication" OR "serial publications" OR brochure* OR leaflet* OR edutainment OR infotainment OR message* OR marketing OR publicity OR "public information" OR "positive framing" OR poster OR posters OR billboard* OR visual art OR street art OR spokesperson* OR celebrit* OR "public relations" OR "Mass Media" OR "Mass Medium" OR internet OR "social media" OR radio OR television OR Newspapers OR Videotape OR "Motion Pictures" OR Telecommunication OR Videorecording OR "Audiovisual Aids" OR "audiovisual equipment" OR facebook OR twitter OR youtube OR "soap opera" OR blog* OR instagram OR cinema* OR movie* OR film* OR magazin* OR magasin* OR phone* OR telephone* OR electronic mail* OR email* OR "e-mail*" OR webmail* OR "mailing list*" OR "discussion list*" OR listserv* OR phone* OR telephone* OR texting OR texted OR sms OR mms OR audio* OR video OR videos OR multimedia OR "multi media" OR web OR website* OR online OR weblog* OR podcast* OR portal* OR "computer mediated" OR apps OR tweet OR bebo OR myspace OR chatroom OR chatrooms OR "viral message" OR wiki* OR virtual* OR booklet* OR flyer* OR "printed media" OR "print media" OR "printed material" OR "print material" OR publication* OR festival* OR entertainment OR "distribute information" OR "distributed information" OR "information distribution" OR "disseminate information" OR "awareness raising" OR "raised awareness" OR "raising awareness" OR "awareness raising" OR "community education program" OR "community education programme" OR "broad based education" OR "broadbased education" OR "promotional item" OR "promotional material" OR "communication program" OR "communication programs" OR "communication programme" OR "communication strategy" OR "communication strategies" OR "famous person" OR "famous persons" OR "famous people" OR "social contact" OR testimon*)
#2	SU (Cesarean Section OR OR "normal childbirth" OR "normal birth" OR "vaginal birth" OR "vaginal childbirth" OR "Abdominal Deliveries" OR "C Section" OR "C Sections" OR Postcesarean OR Postcaesarean OR Cesarean OR Caesarean) OR TI (Cesarean Section OR OR "normal childbirth" OR "normal birth" OR "vaginal birth" OR "vaginal childbirth" OR "Abdominal Deliveries" OR "C Section" OR "C Sections" OR Postcesarean OR Postcaesarean OR Cesarean OR Caesarean)
#3	#1 AND #2
Global Index Medicus http://www.globalhealthlibrary.net/php/index.php	
Concept	Search string

#1	(("health education" OR promotion OR "Health Literacy" OR mhealth OR "government publications as topic" OR "information dissemination" OR "Health promotion" OR "mass media" OR "mass medium" OR "mass communication" OR "mass information" OR campaign* OR advert* OR "social marketing" OR broadcast* OR pamphlet* OR "serial publication*" OR brochure* OR leaflet* OR edutainment OR infotainment OR "advertising" OR "persuasive communication" OR message* OR marketing OR publicity OR "public information" OR "positive framing" OR poster OR posters OR billboard* OR "visual art*" OR "street art*" OR "famous persons" OR spokesperson* OR celebrit* OR "public relation*" OR "Communications Media" OR "Mass Media" OR "Mass Medium" OR Internet OR internet OR "social media" OR radio OR television OR TV OR Newspapers OR "tape recording" OR Videotape OR "Motion Pictures" OR Telecommunication OR "video recording" OR Videorecording OR "Audiovisual Aids" OR "audiovisual equipment" OR facebook OR twitter OR youtube OR ICT OR "soap opera" OR blog* OR instagram OR cinema* OR movie* OR film* OR magazin* OR magasin* OR phone* OR telephone* OR "electronic mail*" OR email* OR e-mail* OR webmail* OR "mailing list*" OR "discussion list*" OR listserv* OR phone* OR telephone* OR text OR texting OR texted OR sms OR mms OR audio* OR video OR videos OR cassette* OR tape* OR dvd* OR "compact dis*" OR cd OR cds OR multimedia OR "multi media" OR web OR website* OR online OR weblog* OR podcast* OR portal* OR e-communication* OR "electronic communication*" OR "computer program*" OR "computer mediated" OR apps OR tweet OR bebo OR myspace OR chatroom OR chatrooms OR "viral message" OR wiki* OR virtual* OR books OR booklet* OR flyer* OR "Printed media" OR "print media" OR "printed material*" OR "print material*" OR publication* OR publications OR festival* OR entertainment) OR(("community intervention" OR ("Community Networks") OR ("Community Health Services")) AND Media) OR (information AND (distribute OR distributed OR distribution OR disseminat*)) OR ((raise OR raised OR raising) AND awareness) OR ((community OR broadbased OR "broad based" OR public) AND "education program*") OR (promotion* AND (item OR items OR material OR materials)) OR (communication AND (program* OR strategy OR strategies)) OR (famous AND (person OR persons OR person's OR people)) OR ((virtual OR indirect OR record OR records OR recording* OR film OR films OR filmed OR audio*) AND ("social contact" OR testimon* OR story OR story's OR stories OR account OR experience* OR narrative* OR play OR ethnograph*)))
#2	("Natural Childbirth" OR "normal childbirth" OR "normal birth" OR "vaginal birth" OR "vaginal childbirth" OR "Cesarean Section" OR "Abdominal Deliveries" OR "C Section" OR "C Sections" OR Postcesarean OR Postcaesarean OR Cesarean OR Caesarean)
#3	#1 AND #2

SCOPUS DATABASE<br>https://www.scopus.com/	
Concept	Search string
#1	"health education" OR promotion OR "Health Literacy" OR mhealth OR "government publications as topic" OR "information dissemination" OR "Health promotion" OR "mass media" OR "mass medium" OR "mass communication" OR "mass information" OR campaign*
#2 -	("Natural Childbirth" OR "normal childbirth" OR "normal birth" OR "vaginal birth" OR "vaginal childbirth" OR "Cesarean Section" OR "Abdominal Deliveries" OR "C Section" OR "C Sections" OR Postcesarean OR Postcaesarean OR Cesarean OR Caesarean)
#3	#1 AND #2
