# Supplementary material for: Mass media campaigns to reduce unnecessary caesarean sections: a systematic review
Source: BMJ Glob Health. 2020 Feb 26;5(2):e001935. doi: 10.1136/bmjgh-2019-001935 (PMC7146028; doi:10.1136/bmjgh-2019-001935)
Supplement: Supplementary data [file bmjgh-2019-001935supp002.pdf]

# Supplementary file 2. Data extraction form

| |
|---|
| **1. Identification (ID number** |
| author, year, reference (journal or website link) |
| Name of the campaign |
| **2. Design of study (RCT, CBA, ITS, Other)** |
| **3. Setting and period of campaign** |
| Country/ region/ city where campaign was run |
| In which languages was the campaign conducted? |
| Start and End dates of campaign, Duration |
| Phases/ additional info/ observations |
| **4. Target population of campaign** |
| **5. Creation / production of campaign** |
| Use of theory of health behavior or behavior change or other theory? |
| Was there any prior scientific work to inform the design of the campaign? |
| Main group/person coordinating the campaign |
| Were government authorities/ personnel involved? |
| Were NGOs or other organizations involved? |
| Were professional media experts/communicators involved in design of the campaign? |
| Were psychologists or experts in mass behavior involved in design of the campaign? |
| **6. Funding for campaign** |
| Main funder and others |
| Total cost of campaign |
| Other information about funding |
| **7. Objectives of campaign** |
| a) Main objectives of campaign (as described by original authors/creators |
| b) Other objectives |
| c) Did campaign have a specific quantitative target? (NI, N, Y-specify) |
| **8. Type of intervention (isolated or part of multicomponent intervention)** |
| **9. Main messages of campaign (as described by authors):** |
| **10. Communication strategy** |
| a) Did it involve **direct communication** with the target population? Specify details |
| b) Did it involve **indirect communication** with the target population? Specify details |
| c) Did the campaign have **spokesperson(s)/celebrity(ies)**? Specify details |
| d) Any **additional relevant communication details** of campaign |
| **11. Outcomes of campaign (impact)** |
| Total N of persons exposed to the campaign |
| How was this number measured/estimated? |
| a) **Changes in Rates of CS** or VD (before and after campaign) measured? |
| N participants assessed before and N assessed after campaign |
| Main characteristics of participants |
| Source of info (birth certificates, survey, other describe): |
| Results: rates CS (before and after campaign) |
| Period when data was collected (years, months before and after campaign) |
| b) **Changes in Preferences for CS** (before and after campaign) measured? |
| N participants assessed before and N assessed after campaign |

| |
|---|
| Main characteristics of participants |
| Type of instrument used (questionnaire/ interviews, other methods) |
| Results: preference for CS (before and after campaign) |
| When was data was collected? |
| c) **Changes in Knowledge about risks/benefits of CS** (before x after campaign) measured? |
| N participants assessed before and N assessed after campaign |
| Main characteristics of participants |
| Type of instrument used (questionnaire/ interviews, other methods) |
| Results: knowledge about risks/benefits of CS (before and after campaign) |
| When was data was collected? |
| d) **Other outcomes** (specify which and give details on how and when assessed) |
| **12. Methods for outcome assessment** |
| Did authors calculate sample size to assess outcome(s)? |
| Were outcome assessors (after campaign) blinded to participants´ exposure to campaign? |
| Any other information about methodological aspects for outcome assessment |
| **13. Main Barriers to create and implement campaign (described by authors)** |
| **14. Main Enablers to create and implement campaign (describe below)** |
| **15. Additional information/ observations** |
