# Supplementary material for: Mass media campaigns to reduce unnecessary caesarean sections: a systematic review
Source: BMJ Glob Health. 2020 Feb 26;5(2):e001935. doi: 10.1136/bmjgh-2019-001935 (PMC7146028; doi:10.1136/bmjgh-2019-001935)
Supplement: Supplementary data [file bmjgh-2019-001935supp003.pdf]

Supplementary file 3. List of excluded studies

	Study	Reason for exclusion
1	Agoratus, Lauren. Improving Pregnancy Outcomes For Families: MODEL PROGRAMS OF THE SPAN PARENT ADVOCACY NETWORK Exceptional Parent 2018;48(8):32-3	Does not describe a mass media campaign
2	Anon. Promocao ao parto normal: uma declaracao conjunta Ministerio da Saude/FEBRASCO/Pastoral da Crianca-CNBB/OMS/UNICEF 1993;():14-14 1993-	Does not describe a mass media campaign
3	Archer, Joseph. 'Normal Birth' Campaign Axed. The Sun, 2017;():20-20	Does not describe a mass media campaign
4	Bainbridge, J. The National Childbirth Trust: 50 years of supporting natural birth British Journal of Midwifery 2006;14(7):416-416	Does not describe a mass media campaign
5	Barnes, M. The power of user pressure. AIMS Journal 2009;21(1):11-11	Does not describe a mass media campaign
6	Battilomo, S. To be born today in Italy: The role of the ministry of health Italian Journal of Pediatrics 2017;43	Does not describe a mass media campaign
7	Behzadifar, M.; Bakhtiari, A.; Azari, S.; Saki, M.; Golbabayi, F.; Bragazzi, N. L. The effect of the health transformation plan on cesarean section in Iran: a systematic review of the literature. BMC research notes 2019;12(1):37	Does not describe a mass media campaign
8	Bingham, Debra; Ruhl, Catherine; Cockey, Carolyn Davis. Don't Rush Me ... Go the Full 40: AWHONN's Public Health Campaign Promotes Spontaneous Labor and Normal Birth to Reduce Overuse of Inductions and Cesareans JOURNAL OF PERINATAL EDUCATION 2013;22(4):189-193	Different objective of the campaign
9	Brody, Jane E. A Campaign to Carry Pregnancies to Term. New York Times 2011;160(55492):7	Different objective of the campaign
10	Calixto Gomes, Samara; Parente Pinheiro Teodoro, Lívia; Alves Pinto, Antonio Germane; Rakelly de Oliveira, Dayanne; da Silva Quirino, Glauberto; Bezerra Pinheiro, Ana Karina. Renascimento do parto: reflexões sobre a medicalização da atenção obstétrica no Brasil. Revista Brasileira de Enfermagem 2018;71(5):2744-2748	Does not describe a mass media campaign
11	Calixto Gomes, Samara; Parente Pinheiro Teodoro, Lívia; Germane Alves, Antonio; Rakelly de Oliveira, Dayanne; da Silva Quirino, Glauberto; Bezerra Pinheiro, Ana Karina. Rebirth of childbirth: reflections on medicalization of the Brazilian obstetric care Revista Brasileira de Enfermagem 2018;71(5):2594-2598	Does not describe a mass media campaign
12	Camm, J. Hospital Trust supports the RCM 'Campaign for normal birth'. RCM midwives : the official journal of the Royal College of Midwives 2005;8(11):443	Does not describe a mass media campaign
13	Campaign to cut the caesarean rate from one in four is failing. Daily Mail 2009;():7	Does not describe a mass media campaign
14	Darsareh F, Aghamolaei T, Rajaei M, Madani A, Zare S. B Butterfly Campaign: A social marketing campaign to promote normal childbirth among first-time pregnant women. Women Birth. 2019 Apr;32(2):e166-e172	Does not describe a mass media campaign
15	Day-Stirk, F. The big push for normal birth. RCM midwives : the official journal of the Royal College of Midwives 2005;8(1):18-20	Campaign not directed at lay people
16	Downe, S. Campaign for Normal Birth. Confidence through support. RCM midwives : the official journal of the Royal College of Midwives 2007;10(8):391	Campaign not directed at lay people
17	Downe, S. Campaign for normal birth. Trust and expertise RCM midwives : the official journal of the Royal College of Midwives 2007;10(2):66	Campaign not directed at lay people
18	Flashenberg, Debra. CELEBRITY BIRTHS: The Good, the Bad, the influence. Midwifery Today 45-45 2011 Spring;(97):45.	Does not describe a mass media campaign

19	Fletcher, G. RCM 'Campaign for normal birth' and the NCT: helping women fight fear of childbirth RCM Midwives 2006;9(2):63-63	Campaign not directed at lay people
20	Fox, S. Campaign for normal birth. Angharad's story. RCM midwives : the official journal of the Royal College of Midwives 2007;10(1):31	Campaign not directed at lay people
21	Fox, S. Campaign for normal birth. RCM midwives : the official journal of the Royal College of Midwives 2007;10(7):319 2007	Campaign not directed at lay people
22	Fraser, D. Campaign for normal birth. Recording facts. RCM midwives : the official journal of the Royal College of Midwives 2007;10(9):432	Campaign not directed at lay people
23	Fraser, D. Campaign for Normal Birth. Students' stories. RCM midwives : the official journal of the Royal College of Midwives 2007;10(3):113	Campaign not directed at lay people
24	Goer, H. The assault on normal birth: the OB disinformation campaign. Midwifery today with international midwife 2002;(63):10-14	Does not describe a mass media campaign
25	Gould, D. Rising caesarean section rates: the power of mass suggestion. British Journal of Midwifery 2007;15(7):398-398	Does not describe a mass media campaign
26	Grytten, J.; Skau, I.; Sørensen, R. The impact of the mass media on obstetricians' behavior in Norway. Health Policy 2017;121(9):986-993	Does not describe a mass media campaign
27	Hadjigeorgiou, E.; Spyridou, A.; Christoforou, A.; Iannuzzi, L.; Giovinale, S.; Canepa, M. M.; Morano, S.; Jonsdottir, S. S.; Karlsdottir, S. I.; Downe, S. Variation in caesarean section rates in Cyprus, Italy and Iceland: an analysis of the role of the media Minerva ginecologica 2018;70(6):676-686	Does not describe a mass media campaign
28	Hales, Katherine. MEDIA AWARENESS: Response to recent reports. Midwifery Matters 2017;(154):14-14	Does not describe a mass media campaign
29	Hauck, Flávia Terra. Implantação da assistência humanizada ao parto em Juiz de Fora (1998-2001).2013;():88-88	Does not describe a mass media campaign
30	Henry, Vaughan. Midwives to end campaign for 'normal' births. Independent (UK) 2017;	Does not describe a mass media campaign
31	International Business, Times. Top 5 Tips and Celebrities Who Gave Birth by C-Section. International Business Times	Does not describe a mass media campaign
32	Johnston, D.; Harman, J. Campaign for normal birth. Beware of institutional defences!. RCM midwives : the official journal of the Royal College of Midwives 2007;10(10):476	Does not describe a mass media campaign
33	Kabakian-Khasholian, T. Promoting women's participation in maternity care: Examples from Lebanon. International Journal of Gynecology and Obstetrics 2012;119():S203	Does not describe a mass media campaign
34	Kennedy, H. P. 'Gone with the Wind' -- interpreting media representations of normal childbirth. International Midwifery 2008;21(1):12-12	Does not describe a mass media campaign
35	Lewitt, MaryJane Promoting Normal Physiologic Birth through Partnership with Consumers, Providers,and Hospitals...Proceedings of the 2015 AWHONN Convention JOGNN: Journal of Obstetric, Gynecologic & Neonatal Nursing 2015;44():S21-S21	Does not describe a mass media campaign
36	Luce, A.; Cash, M.; Hundley, V.; Cheyne, H.; van Teijlingen, E.; Angell, C. "Is it realistic?" the portrayal of pregnancy and childbirth in the media. BMC pregnancy and childbirth 2016;16():40	Does not describe a mass media campaign
37	Michigan Campaign Aims to Reduce Cesarean Deliveries. Medical Economics 2012;89(1):21-21	Does not describe a mass media campaign
38	Morgan, W.; Dankwa-Smith, H. Radio soap operas for family health promotion: a manual for scriptwriters.draft 1994;():140 p. [Unpublished]	Does not describe a mass media campaign
39	Ondeck, Michele. Reflections on the Push for Your Baby Campaign. JOURNAL OF PERINATAL EDUCATION 2012;21(4):206-208	Different objective of the campaign

40	Oshiro BT, Kowalewski L, Sappenfield W, Alter CC, Bettegowda VR, Russell R, Curran J, Reeves L, Kacica M, Andino N, Mason-Marti P, Crouse D, Knight S, Littlejohn K, Malatok S, Dudley DJ, Berns SD. A multistate quality improvement program to decrease elective deliveries before 39 weeks of gestation. Obstet Gynecol. 2013 May;121(5):1025-31.	Different objective of the campaign
41	Queenan, J. T. How to stop the relentless rise in cesarean deliveries. Obstetrics and gynecology 2011;118(2 Pt 1):199-200	Does not describe a mass media campaign
42	Riahi, M.; Taleghani, Y. M.; Vejdani, M.; Salehiniya, H.; Fadae, A. Health sector evolution plan and propagating natural childbirth in Iran. Research Journal of Pharmaceutical, Biological and Chemical Sciences 2017;8(1):58-63	Does not describe a mass media campaign
43	Ruhl, Catherine; Cockey, Carolyn D. 'Don't Rush Me.Go the Full 40' as a Public Health Strategy to promote Spontaneous Labor and Normal Birth JOGNN: Journal of Obstetric, Gynecologic & Neonatal Nursing 2014;43(Supp 1):S24-S25	Does not describe a mass media campaign
44	Sakala, C.; Mayberry, L. J. Vaginal or cesarean Birth? Application of an advocacy organization-driven research translation model Nursing Research 2006;55(2 SUPPL. 1):S68-S74	Does not describe a mass media campaign
45	Silva, H. M.; Ribeiro, C. D.; Lara, L. A. D. S. Cesarean section as the final outcome of pregnancy: The media's influence on the preference of pregnant women through delivery Journal of Sexual Medicine 2011;8():238	Does not describe a mass media campaign
46	Silva, Marina Maria Ribeiro Gomes da Parir é natural RECIIS (Online) 2016;10(2):1-6 2016 .The power of user pressure : MSLC chair Michelle Barnes campaigns successfully to reduce the caesarean rate in Sheffield. AIMS Journal 2009;21(1):11- duplicate of Barnes M. The power of user pressure	Does not describe a mass media campaign
47	Sinclair, M. Campaign for normal birth. Creating good birth memories: personal reflections. RCM Midwives 2007;10(11):524-524	Does not describe a mass media campaign
48	Sinclair, M.; Boreland, Z.; McCabe, N. Campaign for normal birth: less intervention. RCM midwives : the official journal of the Royal College of Midwives 2007;10(5):210	Does not describe a mass media campaign
49	Velasco Juez, Ma Casilda. The Spanish Midwives Association Federation promoted physiological processes in reproductive health: Normal Childbirth Initiative ;():59-62	Does not describe a mass media campaign
