# Supplementary figures and images for: Mass media campaigns to reduce unnecessary caesarean sections: a systematic review
Source: BMJ Glob Health. 2020 Feb 26;5(2):e001935. doi: 10.1136/bmjgh-2019-001935 (PMC7146028; doi:10.1136/bmjgh-2019-001935)

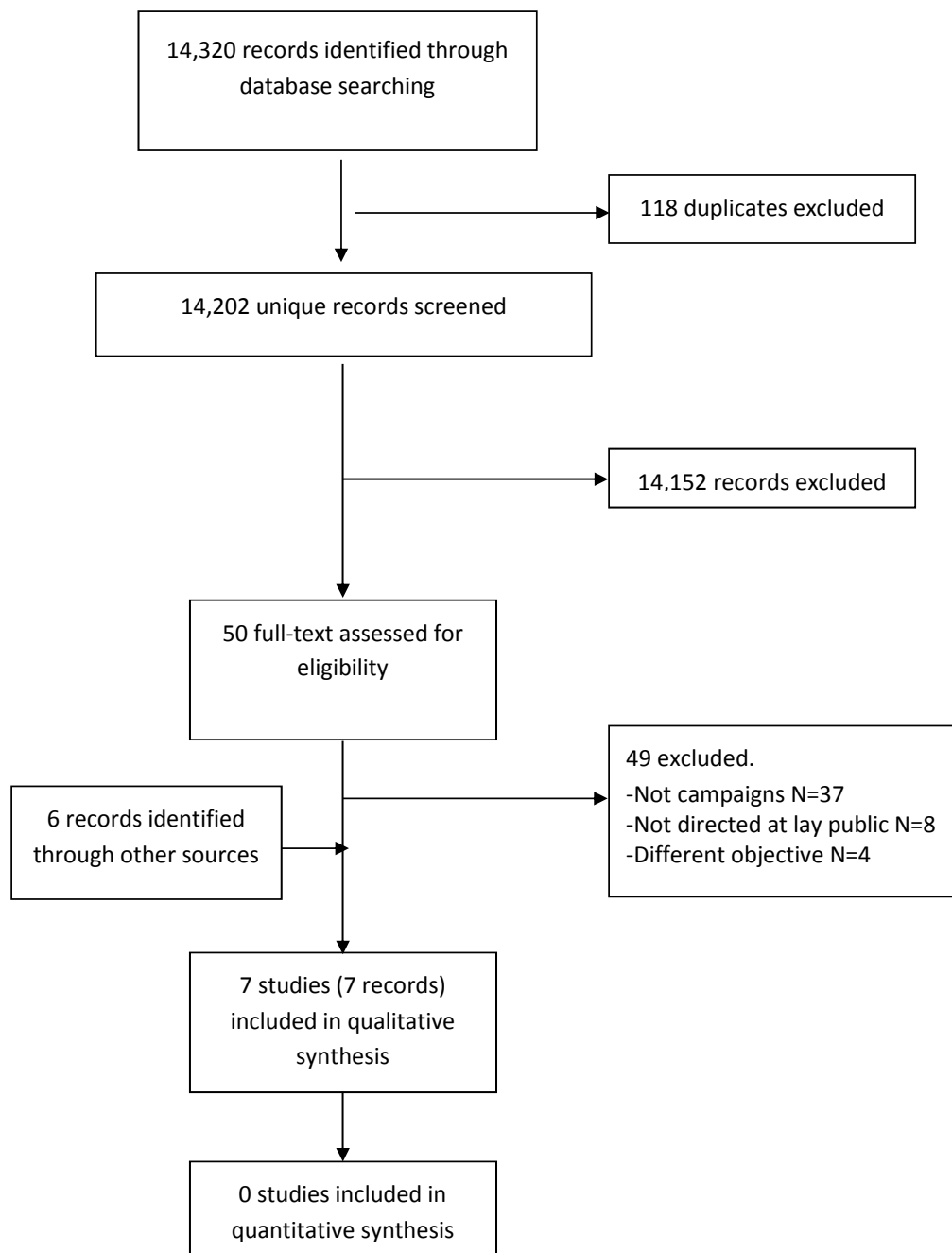

Supplement: Supplementary data [file bmjgh-2019-001935supp004.pdf]
